# Embryonic viability, lipase deficiency, hypertriglyceridemia and neonatal lethality in a novel LMF1-deficient mouse model

**DOI:** 10.1186/1743-7075-11-37

**Published:** 2014-08-16

**Authors:** Nicole Ehrhardt, Candy Bedoya, Miklós Péterfy

**Affiliations:** 1 Medical Genetics Research Institute, Cedars-Sinai Medical Center, Los Angeles, CA 90048, USA; 2 Department of Biomedical Sciences, Cedars-Sinai Medical Center, Los Angeles, CA 90048, USA; 3 Department of Medicine, David Geffen School of Medicine, University of California, Los Angeles, CA 90095, USA

**Keywords:** Lipase Maturation Factor 1, LMF1, Lipase deficiency, Hyper-triglyceridemia

## Abstract

**Background:**

Lipase Maturation Factor 1 (LMF1) is an ER-chaperone involved in the post-translational maturation and catalytic activation of vascular lipases including lipoprotein lipase (LPL), hepatic lipase (HL) and endothelial lipase (EL). Mutations in LMF1 are associated with lipase deficiency and severe hypertriglyceridemia indicating the critical role of LMF1 in plasma lipid homeostasis. The currently available mouse model of LMF1 deficiency is based on a naturally occurring truncating mutation, *combined lipase deficiency* (*cld*), which may represent a hypomorphic allele. Thus, development of LMF1-null mice is needed to explore the phenotypic consequences of complete LMF1 deficiency.

**Findings:**

In situ hybridization and qPCR analysis in the normal mouse embryo revealed ubiquitous and high-level LMF1 expression. To investigate if LMF1 was required for embryonic viability, a novel mouse model based on a null-allele of LMF1 was generated and characterized. LMF1-/- progeny were born at Mendelian ratios and exhibited combined lipase deficiency, hypertriglyceridemia and neonatal lethality.

**Conclusion:**

Our results raise the possibility of a previously unrecognized role for LMF1 in embryonic development, but indicate that LMF1 is dispensable for the viability of mouse embryo. The novel mouse model developed in this study will be useful to investigate the full phenotypic spectrum of LMF1 deficiency.

## Findings

### Introduction

Lipoprotein lipase (LPL), hepatic lipase (HL) and endothelial lipase (EL) are extracellular lipases involved in plasma lipid homeostasis [1]. The secretion and activity of these lipases depends on their post-translational maturation within the endoplasmic reticulum (ER) of lipase-expressing cells [2]. The maturation of lipases involves folding and assembly of nascent polypeptides into catalytically active homodimers, a hallmark structural feature shared among LPL, HL and EL [3]. We have previously demonstrated that lipase maturation factor 1 (LMF1), an ER membrane protein, plays a critical role in lipase maturation [4,5]. In the absence of LMF1, lipases fail to mature into active enzymes, are retained within the ER and undergo intracellular degradation [3]. Highlighting the critical role of LMF1 in lipase expression and lipid metabolism, patients with homozygous LMF1 mutations exhibit plasma lipase deficiency and hypertriglyceridemia [5-7].

LMF1 was initially identified as the gene disrupted by ‘*combined lipase deficiency’* (*cld*), a naturally occurring mutation in the mouse [5]. Homozygous *cld* mutant mice exhibit deficiency in plasma LPL and HL activity, develop hyperchylomicronemia and die within a few days after birth likely due to circulatory problems [8]. While the *cld* mouse model has proven valuable for the identification of LMF1, it has several limitations. First, owing to the presence of a recessive lethal mutation (T, brachyury) *in trans* to the *cld* allele, “wild-type” (+/+) progeny obtained from *cld*/+breeders are not viable, which precludes the use of +/+littermate controls in phenotypic analysis [9]. Second, the *cld* mutation arose on an outbred genetic background harboring a variant form of chromosome 17, known as the *t*-haplotype, which is enriched in deleterious mutations affecting several nearby genes [9]. Consequently, phenotypes observed in *cld* mice cannot be unequivocally ascribed to LMF1 deficiency. Finally, the *cld* allele produces a truncated form of LMF1 (LMF1^cld^) corresponding to ~60% of the full-length protein. As LMF1^cld^ is stable and correctly localizes to the ER membrane, *cld* may represent a hypomorphic allele [4]. In conclusion, characterization of the full phenotypic spectrum of LMF1-deficiency requires the generation of mice with a null-allele of LMF1 on an inbred genetic background.

The present study was triggered by the unexpected observation of widespread LMF1 expression in the developing mouse embryo. This observation raised the possibility that LMF1 may be required for embryonic viability, which may not be apparent in the hypomorphic *cld* model. To test this hypothesis, we generated and characterized a novel mouse model of complete LMF1 deficiency. Our results demonstrate that whereas LMF1 is dispensable for the viability of mouse embryo, LMF1 deficiency is associated with lipase deficiency, hypertriglyceridemia and neonatal lethality. The mouse model developed in this study will facilitate the further characterization of LMF1 in development and metabolic regulation.

## Methods

### Animals

LMF1-deficient mice were generated from a gene-trapped 129/SvEvBrd Omnibank ES cell clone (OST195742) at the Texas A&M Institute for Genomic Medicine [10]. The trapped allele (referred to as ‘LMF1-‘ from here on) was transferred to the C57BL/6J background by backcrossing (N4). Primers used for genotyping (wild-type allele: TG0048-5’ and TG0048-3’; gene-trap allele: LTR2 and KO-A) are listed in Table 1. Mice were maintained on standard laboratory chow (LabDiet 5001) in a specific pathogen-free facility under 14:10 hour light cycle. All animal studies were approved by the Institutional Animal Care and Use Committee at Cedars-Sinai Medical Center.

**Table 1:** Primer sequences

**Name**	**Sequence**	**Application**
TG0048-3'	AGGCTAAGACTCTTTAGGCTCAGG	Genotyping
TG0048-5'	GGCGAGACGATGCTAATTCTATTCC	Genotyping
LTR2	AAATGGCGTTACTTAAGCTAGCTTGC	Genotyping
KO-A	CCGCTTTTCCTGAAGTGAAGGA	Genotyping
T7-LMF1	GCCAGTAATACGACTCACTATAGGGATGCGCCCAGACAGCCTAGTA	IVT
T3-LMF1	GCCAGAATTAACCCTCACTAAAGGGATCTGCAGCACTCCAT	IVT
p1	ATGCGCCCAGACAGCCTAGT	RT-PCR
p2	GAGAACCTGCGTGCAATCCAT	RT-PCR
p3	CCCAGAGGGCAGTCATGA	RT-PCR
p4	GGGCATCTCGTCCTTTGT	RT-PCR
p5	GTGACCTGTGCAGGTAGT	RT-PCR
p6	CGTGGAGCTTCTTGTGCCT	qPCR
p7	GGATCTGCAGCACTCCAT	qPCR
36B4-f	CACTGGTCTAGGACCCGAGAAG	qPCR
36B4-r	GGTGCCTCTGGAGATTTTCG	qPCR

### RNA analysis

**Figure 1:**
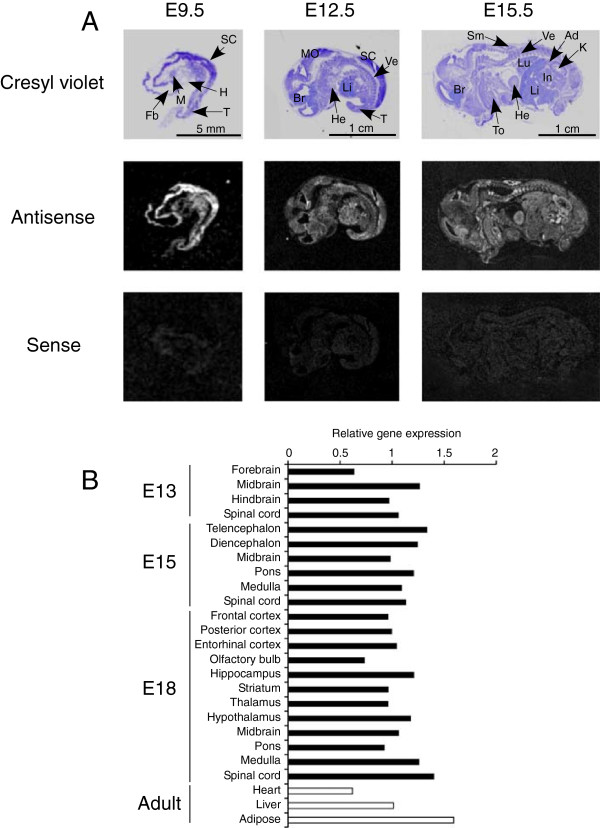
**LMF1 expression in the mouse embryo. (A)***In situ* hybridization of embryo sections with probes representing anti-sense (middle panels) and sense (bottom panels) transcripts of LMF1 at different days *post coitus*. The top panels show bright-field images of cresyl violet-stained sections. Ad, adrenal; Br, brain; Fb, forebrain; H, heart primordium; He, heart; In, intestine; K, kidney; Li, liver; Lu, lung; M, mandibular component of branchial arch; MO, medulla oblongata; SC, spinal cord; Sm, skeletal muscle; T, tail; To, tooth primordium; Ve, vertebrae. **(B)** Real-time PCR analysis of relative LMF1 expression in embryonic and adult tissues.

**Figure 2:**
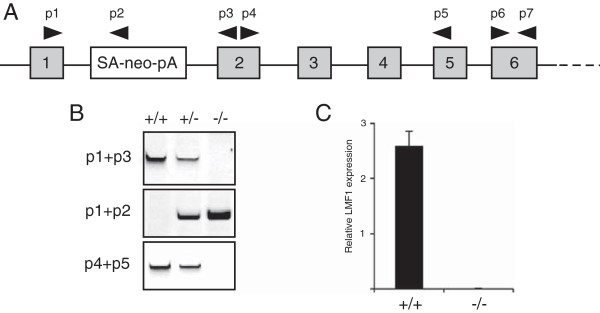
**Generation of LMF1-/- mice. (A)** Schematic illustration of the novel LMF1 allele showing a gene-trap insertion event in intron 1. Gray numbered boxes represent exons. The gene-trap cassette contains a splice acceptor sequence (SA), neomycin resistance gene (neo) and polyadenylation sequence (pA). Arrowheads indicate primers (p1-p5) used for RT-PCR analysis. **(B)** RT-PCR analysis of LMF1 expression in neonatal liver RNA. **(C)** qPCR analysis of LMF1 expression in mouse embryonic fibroblasts using primers p6 and p7 shown in panel A.

*In situ* hybridization was carried out by Phylogeny Inc. as described [11]. ^35^S-UTP-labeled sense and anti-sense cRNA probes were generated by *in vitro* transcription (Ambion) from a T7 and T3 promoter-appended PCR product (primers T7-LMF1 and T3-LMF1 in Table 1) representing the 5’-terminal 889 bp of the LMF1 open reading frame. RNA and cDNA from adult tissues was prepared as described previously [12] and cDNA from embryonic tissues was obtained from OriGene (TissueScan, MDRT). Real-time PCR was performed using LMF1 (#4351372, Life Technologies) and GAPDH (#4352339E) TaqMan assays (Figure 1B), or SybrGreen assays using primers for LMF1 (p6 and p7 in Table 1) and 36B4. For semi-quantitative RT-PCR analysis of the gene-trapped LMF1 allele, primers (p1-p5 in Table 1) spanning multiple exons were used as shown in Figure 2A.

### Plasma assays

To obtain post-heparin plasma from newborn mice, pups were injected intraperitoneally with 10 units of heparin followed by decapitation and blood collection 20 minutes later. Blood was centrifuged (4,500 × g for 5 min) in heparinized separation tubes and plasma was used to determine lipid levels, and enzymatic activities of LPL and HL as described [12]. For the assessment of plasma lipoprotein distribution, plasma obtained from 1-day-old pups was pooled and analyzed by FPLC at the Mouse Metabolic Phenotyping Center at Vanderbilt University School of Medicine. Because of high triglyceride content and viscosity, plasma samples from LMF1-/- animals were diluted 30-fold before FPLC analysis. At this dilution, cholesterol concentrations in fractions could not be reliably determined.

### Data analysis

Results are shown as means ± S.E. Statistical analyses were performed with the SigmaStat software. Two-tailed unpaired Student’s t-test was used to compare means, and chi-square test was applied for the analysis of genotype frequencies. A p-value <0.05 was considered statistically significant.

## Results

### LMF1 expression in the developing mouse embryo

Previous studies indicated that LMF1 was ubiquitously expressed in adult mouse tissues [5]. To investigate whether the broad expression pattern of LMF1 extends to embryonic tissues, we performed *in situ* hybridization studies in the mouse embryo at different stages of development (Figure 1A). In E9.5 mid-gestation embryos, strong LMF1 expression was detected in rudiments of the central nervous system, whereas lower mRNA levels were observed in other tissues. LMF1 exhibited ubiquitous expression pattern in E12.5 and E15.5 embryos with elevated levels in skeletal muscles, cartilage of vertebrae and intestine at late-gestation. To characterize LMF1 expression in the developing CNS further, we performed quantitative RT-PCR analysis on RNA isolated from distinct anatomical regions (Figure 1B). LMF1 showed uniform and ubiquitous expression in all regions and developmental stages tested. Furthermore, LMF1 mRNA levels were comparable to those in principal lipase-expressing adult tissues including adipose, heart and liver (Figure 1B).

### Generation of LMF1-deficient mice

To explore the role of LMF1 in embryonic viability, we generated LMF1-deficient mice using an ES cell clone from a gene-trap library [10]. The targeted allele harbors a gene-trap cassette inserted within the first intron of the LMF1 gene and is predicted to produce a truncated transcript containing exon 1 (Figure 1A). Indeed, transcripts corresponding to exon 1 and the gene-trap cassette were readily detectable in mice carrying the mutant allele (Figure 1B, middle panel). Importantly, sequences 3’ of exon 1 were undetectable in LMF1-/- mice (Figure 1B, top and bottom panels). Moreover, expression of exon 6 could not be detected in LMF1-/- mouse embryonic fibroblasts by qPCR, which confirms the absence of LMF1 transcripts with sequences downstream of the gene-trap integration site (Figure 1C). As exon 1 encodes the N-terminal 64 residues of LMF1, which lacks membrane-spanning domains and corresponds to ~10% of the full-length polypeptide, we conclude that the gene-trap allele most likely represents a null-allele of LMF1.

### Characterization of LMF1-deficient mice

**Figure 3:**
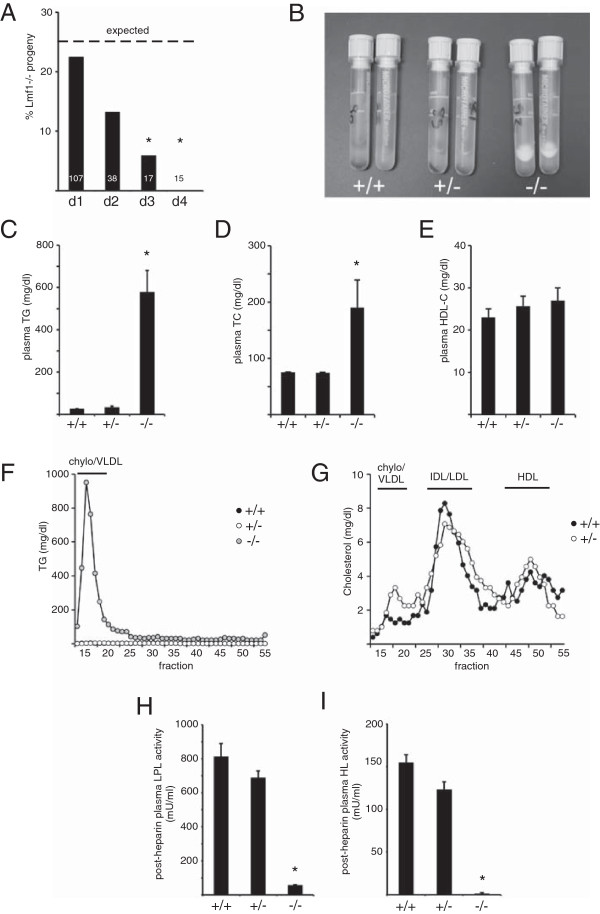
**Characterization of LMF1-/- mice. (A)** Survival of LMF1-deficient (LMF1-/-) progeny during days 1 to 4 (d1 to d4) after birth. Dashed line indicates Mendelian frequency (25%) of -/- genotype as expected from heterozygous matings. White numbers show total number of pups genotyped at each time-point. Asterisks indicate significant (p < 0.05) differences from expected genotype frequencies (χ^2^ test). **(B)** White fat-cake indicates hyperlipidemia in plasma of -/- neonates. **(C)** Plasma triglyceride (TG), **(D)** total cholesterol (TC), and **(E)** HDL-cholesterol (HDL-C) levels are shown in newborn mice. **(F)** Plasma triglyceride and **(G)** cholesterol profiles were determined by FPLC analysis. **(H)** Post-heparin LPL and **(I)** HL activities in newborn mice. *, p < 0.05.

To investigate the impact of LMF1 deficiency on embryonic viability, we genotyped offspring obtained from heterozygous LMF1+/- matings. On the first day after birth, the LMF1-/- genotype was represented at a Mendelian ratio (i.e. 25%) among littermates indicating that LMF1 was dispensable for embryonic viability (Figure 3A). However, the proportion of LMF1-/- pups gradually decreased in subsequent days and no surviving LMF1-deficient progeny could be identified 4 days after birth (Figure 3A).

To assess metabolic manifestations of LMF1 deficiency in the LMF1-/- model, we characterized newborn mice within the first day of life. LMF1-deficient neonates did not exhibit apparent morphological defects. However, their plasma had a milky appearance (Figure 3B), a consequence of ~80-fold elevated triglyceride concentration relative to unaffected littermates (Figure 3C). Hypertriglyceridemia in LMF1-/- pups was due to combined lipase deficiency as demonstrated by dramatically reduced post-heparin LPL and HL activities (Figure 3H and I). As expected, triglycerides were overwhelmingly associated with the chylomicron/VLDL fraction in LMF1-/- plasma (Figure 3F). Relative to wild-type and heterozygous littermates, LMF1-/- mice also demonstrated elevated total cholesterol (Figure 3D), but similar HDL-cholesterol levels (Figure 3E). We were unable to directly assess the cholesterol profile in LMF1-/- mice, because severe lipemia and high viscosity necessitated dilutions of samples before FPLC analysis to an extent, which prevented the detection of cholesterol in fractions. Nonetheless, wild-type and heterozygous mice exhibited similar plasma cholesterol distribution (Figure 3G), where cholesterol was predominantly associated with IDL/LDL particles, a characteristic feature of fetal murine lipoprotein profile [13].

## Discussion

In the present study, we generated a novel mouse model of LMF1 deficiency using gene-trap mutagenesis. The new model offers advantages over the naturally occurring *cld* mutant mouse strain used in previous studies [5,8]. First, in contrast to a truncating mutation in the *cld* model, the gene-trap insertion characterized here represents a definitive null-allele and allows analysis of the full phenotypic manifestation of LMF1 deficiency. An additional improvement of the new model over *cld* is that wild-type littermates are viable, which enables characterization of the potential phenotypic consequences of heterozygous LMF1 deficiency. Finally, the gene-trap allele is on an inbred genetic background devoid of the confounding genetic effects associated with the *cld* mutation [9].

We unexpectedly detected ubiquitous and relatively high-level LMF1 expression in the mouse embryo. This observation raised the possibility that in addition to its established role in postnatal lipid metabolism, LMF1 may also be an important factor in embryogenesis. While the role of vascular lipases during development remains poorly characterized, embryonic expression of all three LMF1-dependent lipases (i.e. LPL, HL and EL) has previously been documented [14,15]. To begin to address the potential role of LMF1 in development, we first asked whether LMF1 deficiency affects the viability of embryos. Our results demonstrate that despite widespread expression in the developing embryo, LMF1 is not required for embryonic survival. However, postnatal viability of LMF1-/- pups is severely compromised, as no surviving LMF1-deficient progeny was detected a few days after birth. Neonatal lethality has also been observed in *cld* mice [8] and is thought to be a consequence of circulatory problems associated with severe hyperchylomicronemia, which results from the inability to utilize dietary fat during suckling [16]. Indeed, LMF1-/- pups exhibit hypertriglyceridemia and severely diminished post-heparin LPL and HL activities, hallmark features of LMF1 deficiency in the *cld* mouse model [8]. In addition to hypertriglyceridemia, plasma concentrations of total cholesterol are also elevated in LMF1-/- mice, a likely consequence of diminished catabolism and accumulation of chylomicron particles due to LPL deficiency [17]. In contrast to total cholesterol, HDL-cholesterol levels are unaffected in LMF1-/- plasma. At first glance, this is a surprising observation considering the critical role of LPL in the maturation of HDL particles [18] and severely reduced HDL-cholesterol in LPL-deficient mice [17,19,20]. However, in addition to LPL, LMF1-/- animals are also deficient in active HL and EL [7], lipases that promote HDL catabolism [21-23]. Thus, we propose that unaltered HDL-cholesterol level in LMF1-/- mice is a result of combined lipase deficiency involving lipases with opposite effects on HDL metabolism.

LMF1-deficient mice developed in this work will allow in-depth investigations of the role of LMF1 in development. However, early lethality is a limitation of this model for metabolic studies in the adult organism. Neonatal lethality in LMF1-/- mice is not unique among mouse models of hypertriglyceridemia. LPL-deficient mice die within a few days after birth, most likely as a consequence of restricted oxygen exchange in lipid-engorged lung capillaries [17]. In contrast, hypertriglyceridemia in mice deficient in GPIHBP1, a protein involved in the trans-endothelial transport of LPL, is not associated with increased mortality owing to the availability of a functional pool of LPL in the neonatal liver [24,25]. Two strategies have been used to rescue LPL-deficient mice from neonatal lethality. First, transient expression of LPL through adenoviral gene transfer allowed a small fraction of infected LPL-/- progeny to reach adulthood and enabled metabolic characterization of LPL-deficiency [18,20,26]. We attempted a similar strategy to rescue LMF1-/- pups using adenovirus expressing LMF1, but have been unable to recover adult LMF1-deficient mice (unpublished observation). Importantly, LMF1-deficient mice are not only devoid of active LPL, but also HL and EL, which may result in more severe morbidity relative to LPL-deficiency only and explain why transient expression of LMF1 is insufficient for rescue. Consistent with this explanation, HL/EL double knock-out mice suffer from neonatal lethality [23], which raises the possibility that combined HL/EL-deficiency contributes to mortality in LMF1-/- mice. A second strategy that has been successfully applied to rescue LPL-/- mice is based on transgenic complementation of LPL expression in single tissues such as heart, skeletal muscle and liver [27-29]. A similar approach is currently pursued in our laboratory to rescue LMF1-/- mice using a muscle-specific LMF1 transgene [12].

In conclusion, we validated a new LMF1-deficient mouse model by demonstrating that it recapitulates salient phenotypes of *cld* mutant mice including neonatal lethality, dyslipidemia and combined lipase deficiency. At the same time, our study also confirms that phenotypes previously observed in the *cld* model are genuine consequences of LMF1 deficiency, as opposed to unrelated mutations present in the *cld* genetic background [9]. The fact that our initial characterization of LMF1-/- neonates did not reveal novel phenotypes beyond those already observed in *cld* mice is consistent with the possibility that *cld* represents a null-allele of LMF1. However, more detailed phenotypic characterization will be necessary before a definitive conclusion can be reached. The LMF1-deficient mouse model developed in this study will facilitate further analysis of LMF1 function in development and metabolic regulation.

## Abbreviations

*cld*: ‘combined lipase deficiency’ mutation; CNS: Central nervous system; EL: Endothelial lipase; ER: Endoplasmic reticulum; HDL: High-density lipoprotein; HL: Hepatic lipase; IVF: *in vitro* translation; LDL: Low-density lipoprotein; LMF1: Lipase maturation factor 1; LPL: Lipoprotein lipase; qPCR: quantitative PCR; RT-PCR: Reverse transcription polymerase chain reaction; VLDL: Very low-density lipoprotein.

## Competing interests

The authors declare no conflict of interest.

## Authors’ contributions

NE carried out molecular genetic and in vivo experiments, participated in the design of the study and performed statistical analysis. CB carried out analyses of embryonic LMF1 expression. MP conceived the study, and participated in its design and coordination and drafted the manuscript. All authors read and approved the final manuscript.

## References

[B1] Hasham SN Pillarisetti S Vascular lipases, inflammation and atherosclerosis Clin Chim Acta 2006 372 179 183 10.1016/j.cca.2006.04.020 16765928

[B2] Doolittle MH Peterfy M Mechanisms of lipase maturation Clin Lipidol 2010 5 71 85 10.2217/clp.09.85 20543905 PMC2883275

[B3] Doolittle MH Ehrhardt N Peterfy M Lipase maturation factor 1: structure and role in lipase folding and assembly Curr Opin Lipidol 2010 21 198 203 10.1097/MOL.0b013e32833854c0 20224398 PMC3924775

[B4] Doolittle MH Neher SB Ben-Zeev O Ling-Liao J Gallagher CM Hosseini M Yin F Wong H Walter P Peterfy M Lipase maturation factor LMF1, membrane topology and interaction with lipase proteins in the endoplasmic reticulum J Biol Chem 2009 284 33623 33633 10.1074/jbc.M109.049395 19783858 PMC2785204

[B5] Peterfy M Ben-Zeev O Mao HZ Weissglas-Volkov D Aouizerat BE Pullinger CR Frost PH Kane JP Malloy MJ Reue K Pajukanta P Doolittle MH Mutations in LMF1 cause combined lipase deficiency and severe hypertriglyceridemia Nat Genet 2007 39 1483 1487 10.1038/ng.2007.24 17994020

[B6] Cefalu AB Noto D Arpi ML Yin F Spina R Hilden H Barbagallo CM Carroccio A Tarugi P Squatrito S Vigneri R Taskinen MR Peterfy M Averna MR Novel LMF1 nonsense mutation in a patient with severe hypertriglyceridemia J Clin Endocrinol Metab 2009 94 4584 4590 10.1210/jc.2009-0594 19820022 PMC2819827

[B7] Ben-Zeev O Hosseini M Lai CM Ehrhardt N Wong H Cefalu AB Noto D Averna MR Doolittle MH Peterfy M Lipase maturation factor 1 is required for endothelial lipase activity J Lipid Res 2011 52 1162 1169 10.1194/jlr.M011155 21447484 PMC3090237

[B8] Paterniti JR Jr Brown WV Ginsberg HN Artzt K Combined lipase deficiency (cld): a lethal mutation on chromosome 17 of the mouse Science 1983 221 167 169 10.1126/science.6857276 6857276

[B9] Peterfy M Mao HZ Doolittle MH The cld mutation: narrowing the critical chromosomal region and selecting candidate genes Mamm Genome 2006 17 1013 1024 10.1007/s00335-006-0045-3 17019649

[B10] Zambrowicz BP Abuin A Ramirez-Solis R Richter LJ Piggott J BeltrandelRio H Buxton EC Edwards J Finch RA Friddle CJ Gupta A Hansen G Hu Y Huang W Jaing C Key BW Jr Kipp P Kohlhauff B Ma ZQ Markesich D Payne R Potter DG Qian N Shaw J Schrick J Shi ZZ Sparks MJ Van Sligtenhorst I Vogel P Walke W Wnk1 kinase deficiency lowers blood pressure in mice: a gene-trap screen to identify potential targets for therapeutic intervention Proc Natl Acad Sci U S A 2003 100 14109 14114 10.1073/pnas.2336103100 14610273 PMC283554

[B11] Hu MC Wang YP Mikhail A Qiu WR Tan TH Murine p38-delta mitogen-activated protein kinase, a developmentally regulated protein kinase that is activated by stress and proinflammatory cytokines J Biol Chem 1999 274 7095 7102 10.1074/jbc.274.11.7095 10066767

[B12] Hosseini M Ehrhardt N Weissglas-Volkov D Lai CM Mao HZ Liao JL Nikkola E Bensadoun A Taskinen MR Doolittle MH Pajukanta P Peterfy M Transgenic expression and genetic variation of Lmf1 affect LPL activity in mice and humans Arterioscler Thromb Vasc Biol 2012 32 1204 1210 10.1161/ATVBAHA.112.245696 22345169 PMC3331946

[B13] van Straten EM Huijkman NC Baller JF Kuipers F Plosch T Pharmacological activation of LXR in utero directly influences ABC transporter expression and function in mice but does not affect adult cholesterol metabolism Am J Physiol Endocrinol Metab 2008 295 E1341 E1348 10.1152/ajpendo.90597.2008 18840761

[B14] Semenkovich CF Chen SH Wims M Luo CC Li WH Chan L Lipoprotein lipase and hepatic lipase mRNA tissue specific expression, developmental regulation, and evolution J Lipid Res 1989 30 423 431 2723548

[B15] Lindegaard ML Nielsen JE Hannibal J Nielsen LB Expression of the endothelial lipase gene in murine embryos and reproductive organs J Lipid Res 2005 46 439 444 15576837 10.1194/jlr.M400417-JLR200

[B16] Reue K Doolittle MH Naturally occurring mutations in mice affecting lipid transport and metabolism J Lipid Res 1996 37 1387 1405 8827513

[B17] Weinstock PH Bisgaier CL Aalto-Setala K Radner H Ramakrishnan R Levak-Frank S Essenburg AD Zechner R Breslow JL Severe hypertriglyceridemia, reduced high density lipoprotein, and neonatal death in lipoprotein lipase knockout mice. Mild hypertriglyceridemia with impaired very low density lipoprotein clearance in heterozygotes J Clin Invest 1995 96 2555 2568 10.1172/JCI118319 8675619 PMC185959

[B18] Strauss JG Frank S Kratky D Hammerle G Hrzenjak A Knipping G von Eckardstein A Kostner GM Zechner R Adenovirus-mediated rescue of lipoprotein lipase-deficient mice. Lipolysis of triglyceride-rich lipoproteins is essential for high density lipoprotein maturation in mice J Biol Chem 2001 276 36083 36090 10.1074/jbc.M104430200 11432868

[B19] Coleman T Seip RL Gimble JM Lee D Maeda N Semenkovich CF COOH-terminal disruption of lipoprotein lipase in mice is lethal in homozygotes, but heterozygotes have elevated triglycerides and impaired enzyme activity J Biol Chem 1995 270 12518 12525 10.1074/jbc.270.21.12518 7759497

[B20] Zhang X Qi R Xian X Yang F Blackstein M Deng X Fan J Ross C Karasinska J Hayden MR Liu G Spontaneous atherosclerosis in aged lipoprotein lipase-deficient mice with severe hypertriglyceridemia on a normal chow diet Circ Res 2008 102 250 256 10.1161/CIRCRESAHA.107.156554 18032735

[B21] Busch SJ Barnhart RL Martin GA Fitzgerald MC Yates MT Mao SJ Thomas CE Jackson RL Human hepatic triglyceride lipase expression reduces high density lipoprotein and aortic cholesterol in cholesterol-fed transgenic mice J Biol Chem 1994 269 16376 16382 8206946

[B22] Ishida T Choi S Kundu RK Hirata K Rubin EM Cooper AD Quertermous T Endothelial lipase is a major determinant of HDL level J Clin Invest 2003 111 347 355 10.1172/JCI16306 12569160 PMC151857

[B23] Brown RJ Lagor WR Sankaranaravanan S Yasuda T Quertermous T Rothblat GH Rader DJ Impact of combined deficiency of hepatic lipase and endothelial lipase on the metabolism of both high-density lipoproteins and apolipoprotein B-containing lipoproteins Circ Res 2010 107 357 364 10.1161/CIRCRESAHA.110.219188 20558822 PMC2948973

[B24] Beigneux AP Davies BS Gin P Weinstein MM Farber E Qiao X Peale F Bunting S Walzem RL Wong JS Blaner WS Ding ZM Melford K Wongsiriroj N Shu X de Sauvage F Ryan RO Fong LG Bensadoun A Young SG Glycosylphosphatidylinositol-anchored high-density lipoprotein-binding protein 1 plays a critical role in the lipolytic processing of chylomicrons Cell Metab 2007 5 279 291 10.1016/j.cmet.2007.02.002 17403372 PMC1913910

[B25] Weinstein MM Tu Y Beigneux AP Davies BS Gin P Voss C Walzem RL Reue K Tontonoz P Bensadoun A Fong LG Young SG Cholesterol intake modulates plasma triglyceride levels in glycosylphosphatidylinositol HDL-binding protein 1-deficient mice Arterioscler Thromb Vasc Biol 2010 30 2106 2113 10.1161/ATVBAHA.110.214403 20814015 PMC2959134

[B26] Ding YL Wang YH Huang W Liu G Ross C Hayden MR Yang JK Glucose intolerance and decreased early insulin response in mice with severe hypertriglyceridemia Exp Biol Med 2010 235 40 46 10.1258/ebm.2009.009100 20404017

[B27] Levak-Frank S Hofmann W Weinstock PH Radner H Sattler W Breslow JL Zechner R Induced mutant mouse lines that express lipoprotein lipase in cardiac muscle, but not in skeletal muscle and adipose tissue, have normal plasma triglyceride and high-density lipoprotein-cholesterol levels Proc Natl Acad Sci U S A 1999 96 3165 3170 10.1073/pnas.96.6.3165 10077655 PMC15913

[B28] Levak-Frank S Weinstock PH Hayek T Verdery R Hofmann W Ramakrishnan R Sattler W Breslow JL Zechner R Induced mutant mice expressing lipoprotein lipase exclusively in muscle have subnormal triglycerides yet reduced high density lipoprotein cholesterol levels in plasma J Biol Chem 1997 272 17182 17190 10.1074/jbc.272.27.17182 9202040

[B29] Merkel M Weinstock PH Chajek-Shaul T Radner H Yin B Breslow JL Goldberg IJ Lipoprotein lipase expression exclusively in liver. A mouse model for metabolism in the neonatal period and during cachexia J Clin Invest 1998 102 893 901 10.1172/JCI2912 9727057 PMC508954

